# ‘A change of heart’: Indigenous perspectives from the Onjisay Aki Summit on climate change

**DOI:** 10.1007/s10584-021-03000-8

**Published:** 2021-02-15

**Authors:** Laura Cameron, Dave Courchene, Sabina Ijaz, Ian Mauro

**Affiliations:** 1 Prairie Climate Centre, University of Winnipeg, 599 Portage Ave, Winnipeg, MB R3B 2G3 Canada; 2 Turtle Lodge, Sagkeeng First Nation, PO Box 1267, Pine Falls, MB R0E 1M0 Canada

**Keywords:** Indigenous knowledge, Climate change, Canada, Indigenous-settler relationships

## Abstract

In June 2017, the Turtle Lodge Indigenous knowledge centre convened the Onjisay Aki International Climate Summit, an unparalleled opportunity for cross-cultural dialogue on climate change with environmental leaders and Indigenous Knowledge Keepers from 14 Nations around the world. In collaboration with Turtle Lodge, the Prairie Climate Centre was invited to support the documentation and communication of knowledge shared at the Summit. This process of Indigenous-led community-based research took an inter-epistemological approach, using roundtable discussions within a ceremonial context and collaborative written and video methods. The Summit brought forward an understanding of climate change as a symptom of a much larger problem with how colonialism has altered the human condition. The Knowledge Keepers suggested that, in order to effectively address climate change, humanity needs a shift in values and behaviours that ground our collective existence in a balanced relationship with the natural world and its laws. They emphasized that their diverse knowledges and traditions can provide inspiration and guidance for this cultural shift. This underscores the need for a new approach to engaging with Indigenous knowledge in climate research, which acknowledges it not only as a source of environmental observations, but a wealth of values, philosophies, and worldviews which can inform and guide action and research more broadly. In this light, Onjisay Aki makes significant contributions to the literature on Indigenous knowledge on climate change in Canada and internationally, as well as the ways in which this knowledge is gathered, documented, and shared through the leadership of the Knowledge Keepers.

**Supplementary Information:**

The online version contains supplementary material available at 10.1007/s10584-021-03000-8.

## Introduction

Climate change is a pressing, multi-dimensional issue, which affects all of humanity across our social, economic, and ecological systems. To date, the discourse regarding climate change and how to respond to it has largely been dominated by scientific experts (IPCC 2014). However, in recent decades, there has been increasing recognition of the value and validity of Indigenous knowledges (IK) in western climate policy and research (Green and Raygorodetsky 2010; Maldonado et al. 2016)—the result of decades of Indigenous organizing, ceremony, lobbying, and advocacy. While Indigenous knowledge systems are diverse, complex, and unique, there are some common principles across them, including relationality, interconnectedness, reciprocity, and balance through a relationship with spirit and the land (Battiste and Henderson 2000; Cajete 2000), which many scholars and Indigenous Knowledge Keepers argue are important in the context of understanding and addressing climate change (Courchene 2019; Raygorodetsky 2011; Wildcat 2009). In Canada, there have been increasing partnerships between researchers and Indigenous communities to collaborate on documenting climate impacts and responses and co-producing knowledge to inform policy, as evidenced by numerous journal special issues (e.g. Ford and Furgal 2009; Green and Raygorodetsky 2010; Salick and Ross 2009) and other works in this field (e.g. Cuerrier et al. 2015; Cunsolo Willox et al. 2012; Fox 2004; Gearheard et al. 2011; Pearce et al. 2015; Turner and Clifton 2009).

While this increase in recognition, inclusion, collaboration, and co-creation marks significant progress, much work remains to engage IK to its full extent, recognizing the embodied nature of the knowledge and the leadership of Indigenous peoples (Arora-Jonsson 2017; Watson and Huntington 2014). Though Indigenous and western knowledges are not completely distinct, and plural logics and epistemologies exist within each which in some cases produce overlapping knowledges (Agrawal 1995), a focus here is on the engagement of Indigenous knowledges and epistemologies in relation to western systems and institutions. Where IK is recognized as valuable, it is often seen as a source of observations about the environment to complement western science within academic, policy, or management frameworks (Watson and Huntington 2014), and as a result includes only what researchers consider ‘complementary’ knowledges. Less often is IK recognized and engaged as a complex and dynamic system—with distinct spiritual and spatial dimensions—that is inseparable from the lived experiences of Indigenous peoples (McGregor 2004; Smith 1999). Whyte (2017b) emphasizes that this shallow engagement of IK in research on sustainability overlooks the governance-value of these knowledges as ‘irreplaceable sources of guidance for Indigenous resurgence and nation-building’ (p. 5). There remains a need for a further shift from recognizing IK as a source of empirical observations or ‘facts’ to engaging with it as a living system derived from and embedded within complex lifeways and governance systems (Berkes 2009; Whyte 2017b). Critically, Indigenous scholars assert that IK must be engaged not only to inform western climate solutions but also to principally strengthen Indigenous adaptive capacities and self-determination (Whyte 2017a, 2017b; Wildcat 2009).

To challenge the colonial dynamics of research in this context, researchers must go beyond simple inclusion of IK and towards understanding and defining the problems and approaches to climate change research according to Indigenous worldviews and ways of knowing. To do so, researchers must heed the calls of Indigenous and allied scholars to move away from characterizations of IK as exclusively ‘local’ and ‘traditional’ and meaningfully engage with the philosophical, epistemological, and ontological dimensions of IK (Cameron 2012; McGregor 2009; Watson and Huntington 2014). In order to grapple with this complexity, Murphy (2011) calls for an approach to climate research ‘that moves beyond the bounds of disciplinary knowledges into a space that is overtly *inter-epistemological*’ (p. 492). This research seeks to take an inter-epistemological approach, guided by the community at Turtle Lodge.

### Turtle Lodge and Onjisay Aki

Turtle Lodge International Centre for Indigenous Education and Wellness, located in Sagkeeng First Nation, Manitoba, was founded in 2002 by Anishinaabe Knowledge Keeper Dave Courchene (Nii Gaani Aki Inini – Leading Earth Man) (www.turtlelodge.org). In 2018, the Lodge was named as a central house of knowledge and place of governance by Knowledge Keepers from across Turtle Island (North America) and Abya Yala (South America) (Ijaz 2018). While the Turtle Lodge community has always been dedicated to environmental balance, following the ethic of relationality and stewardship central to an Anishinaabe worldview (Borrows 2006; Johnston 2003; Kimmerer 2014), in recent years they have taken climate change as a focus in much of their work. Driven by the desire to bring forward deeper understandings of Indigenous perspectives and reclaim a place of leadership for Indigenous peoples in public and political discourses on climate change, Elder Courchene and Turtle Lodge began the Onjisay Aki (‘Our Changing Earth’) Initiative. In June 2017, Turtle Lodge convened the Onjisay Aki International Climate Summit, an unparalleled opportunity for cross-cultural dialogue on climate change, led by Indigenous Knowledge Keepers who shared their spiritual and ancestral knowledges through ceremony and discussion. In collaboration with Turtle Lodge, the research team from the Prairie Climate Centre (PCC) was invited to support the documentation, synthesis, and communication of the knowledge and perspectives shared at the Summit.

This research makes valuable contributions to the growing field of Indigenous leadership on climate change, with three broad objectives exploring (1) the ways in which Indigenous peoples in the Canadian Prairies and beyond are experiencing and understanding the challenges of climate change; (2) the role of Indigenous knowledges and traditions in addressing climate change; and (3) the direction for climate action that Indigenous leaders in this region are bringing forward. This work adds to the emerging body of inter-epistemological studies on IK and climate change in Canada and globally, offering insights for approaches that seek to bridge knowledge systems.

## Methods

The research approach was guided by the Turtle Lodge community and designed with the aim to honour the unique epistemological considerations required when documenting Indigenous knowledges, including oral tradition, cultural and ceremonial protocols associated with knowledge exchange, and the embodied nature of IK in this context. The research team—comprising two Turtle Lodge community members and two academics—adopted an integrated methodology based on principles of both Indigenous and community-based research (CBR) methodologies (Koster et al. 2012; Kovach 2009; Smith 1999; Wilson 2008). Stemming from the need to address power imbalances in conventional qualitative research, CBR approaches have emerged and gained popularity in qualitative research with Indigenous communities (Koster et al. 2012; Halseth et al. 2016). Recognizing the history of exploitative relationships in CBR, the research team formalized processes of community leadership at the outset, and adopted six principles central to the methodology: respectful relationships, community leadership, reciprocity, knowledge exchange, action-oriented, and community ownership over knowledge. This collaborative research was rooted in a long-term and ongoing partnership between the research team members, developed and strengthened over many gatherings, ceremonies, and meetings at the Lodge leading up to, during, and following the Onjisay Aki Summit.

The Summit convened 22 leaders, among them 17 Knowledge Keepers and community leaders from Indigenous Nations including the Anishinaabe, Dakota, Dene, Plains Cree, Lakota, Blackfoot, Cherokee/Choctaw, Haida, Pueblo, and Inka (Appendix). While the discussion was led by these Indigenous participants, five non-Indigenous environmental and social justice leaders were also invited to listen, learn, and offer their own contributions to the discussion. With the exception of one participant from Japan, Indigenous participants were from Nations across the Americas. The views shared as discussed herein are reflective of the specific knowledges they hold, and cannot be generalized to all Indigenous peoples. Herein, the term Indigenous is used to refer to people who identify as descendants of the original inhabitants of their ancestral homelands, who have (or had) a unique Indigenous language and identity, and who distinguish themselves from other cultural groups (as discussed by Corntassel 2010).

The roundtable discussion proceeded in the sacred Turtle Lodge over four days, led by traditional ceremonies of Indigenous peoples from across North and South America and following Anishinaabe protocols of a talking circle, facilitated by Chair and Anishinaabe Knowledge Keeper Dr. Dave Courchene. Importantly, talking circles create opportunity for storytelling, an integral part of Indigenous ways of knowing; Indigenous stories are inseparable from knowing; thus, narrative is an essential part of research in Indigenous frameworks (Kovach 2009). The discussions were fully recorded on video, with the exception of the Pipe and Water ceremonies, as per Turtle Lodge protocols. Video recordings were transcribed and transcripts collaboratively qualitatively analysed. Content analysis was conducted with the Summit transcripts, as well as transcripts from two discussions with Elders during the planning of the Summit, to identify common themes, thoughts, stories, and experiences (Baxter 2009). A coding scheme of categories, themes, and sub-themes was developed, refined, and reorganized through three rounds of qualitative coding in a qualitative analysis software, NVivo 11.4 (Miles et al. 2014). Through iterative discussions between researchers and Turtle Lodge community partners, narrative emerged out of the codes and connections among ideas. Frequency analysis of the codes also helped to guide interpretations of the findings. The writing was conducted primarily by the academic members of the research team, with oversight, continuous feedback, and ongoing approval of the Turtle Lodge partners. Simultaneously, several short videos were made with the footage of the Summit through a process of participatory video (Milne et al. 2012), highlighting the main themes and outcomes of the discussion to share with a wider audience.

## Results

Over four days of ceremony and discussion, Knowledge Keepers and environmental leaders discussed causes, impacts, and solutions associated with climate change. The contributions of each participant were unique and diverse, reflecting their distinct Nationhood and cultures, but nonetheless, many points of agreement arose through discussion. In general, the discussion reflected a holistic understanding of climate change, high level insights illustrating the complex and interconnected problems driving the issue, systems of domination such as settler colonialism, capitalism, and imperialism, and the leadership of Indigenous peoples in solutions. Themes and sub-themes that emerged from the analysis were organized into three major categories which are discussed below: causes and impacts of climate change; Indigenous knowledges and traditions; and Indigenous directions for climate action.

### Causes and impacts of climate change: a reflection of values

Themes that emerged from the analysis related to the causes and impacts of climate change were: impacts and environmental changes; colonialism; human condition, values, and behaviours; and general problems. Participants talked about the increasingly visible and devastating impacts of climate change—changes in weather, animal migrations, seasonal duration, water quality and levels, quality of significant plants and medicines, forest fires, and melting icecaps. Many people spoke from their own experiences and observations of changes in their communities over their lifetimes. Chief Jack Caesar from the Ross River Dene in the Yukon Territory described the changes he has experienced:

The weather patterns are really changing up in the north… [they] are really changing the seasons there for us. We’re at a time where it’s getting a lot warmer, there’s less snow... And the soils and the permafrost that we depend on, that too is changing.

People discussed how these environmental changes are impacting the social well-being of their communities—decreasing their ability to practice their traditional livelihoods, decreasing the health and safety of their people, and having significant emotional and psychological impacts resulting from being disconnected from the land. These changes were discussed in relation to other challenges and impacts that communities are experiencing and observing around the world—pollution from extraction and mining, widespread sickness among people and animals, religious extremism, war and violence, gender inequality, poor mental health, and suicide epidemics. There was a general sense in the discussion that humanity is in a time of multiple crises which are all interconnected, and which many see as tied to colonialism.

**Fig. 1:**
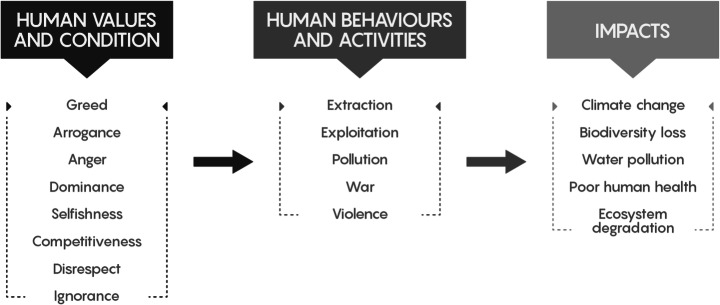
The Elders described elements of the human condition and values, which motivate human behaviours and activities, which cause the impacts that we are facing today

The legacies and ongoing impacts of colonization were a major focus of the discussion, in relation to these social and environmental crises. Elders and Indigenous community leaders spoke of the tools of colonization—such as the Indian Act, the Doctrine of Discovery, and other colonial government legislations attempting to control Indigenous peoples—which they assert must be dismantled, rejected, or changed. Participants discussed how colonial and capitalist systems have brought forward and imposed a set of Eurocentric values, while repressing Indigenous traditional values and ways of knowing. Many Knowledge Keepers described how these Eurocentric values have created a human condition—based on greed, anger, competition, selfishness, arrogance, ignorance, disrespect, and domination—which fuels destructive human behaviour and activities—such as extraction and exploitation of resources, war and violence, and pollution and manipulation of ecosystems (Fig. 1). They explained that these behaviours are working against the natural balance of life—as evidenced through severe impacts such as climate change, biodiversity decline, ecosystem degradation, water pollution and shortage, and poor health of people—increasingly disconnecting people from the land and from each other. Elder Dr. Dave Courchene described this condition from his perspective: ‘climate change is really a reflection of values that are creating this imbalance that we’re finding in today’s world’.

In discussing the negative values and behaviours at the root of the environmental impacts we face today, some participants referred to humanity as ‘lost’ or ‘asleep’ in our actions and our failure to respond to the problems. Others described humanity as being misguided by a desire for power, control, and material wealth. These comments reflect an understanding that the environmental crisis is a human crisis—inextricably linked to racism, violence, trauma, and domination. Dakota Knowledge Keeper Katherine Whitecloud described this personal problem of climate change:

People don’t want to acknowledge the state of the Earth, where it’s at right now, because it’s a reflection of themselves. It’s a reflection of their homes, their personal space, where the spirit and the heart reside… And people don’t want to look at that.

Many Knowledge Keepers also commented on peoples’ failure to listen to the voice and messages of the Earth, and to uphold their responsibilities to reciprocate for the gifts that the Earth has given humanity. Pueblo professor Vivian Delgado described climate change as ‘the wordless way that the Earth speaks to us’. Similarly, Elder Dr. Dave Courchene described the changes in the Earth as ‘the way that nature can teach us to realize the impacts of our actions’. There was an understanding that the Earth is alive and has an intelligence that is guiding humanity to respond to these changes. The late Anishinaabe Elder Elmer Courchene questioned: ‘Are we paying attention to what nature is telling us? Are we listening to the winds, are we listening to the forest, are we listening to the waters, to the thunders?’ There was a common understanding that, because of peoples’ disconnection from the land, they are not able to hear and understand the messages that the changes are bringing, and to act in response.

### Indigenous knowledges and traditions: a foundation for addressing climate change

From an understanding that climate change is driven by colonial behaviours and values, most participants suggested that people reconnecting with their knowledges and traditions must be the foundation for climate action, led by Indigenous peoples and inclusive of all people. Participants discussed the ways in which Indigenous ways of being and knowing can provide guidance for solutions to the imbalance in the environment and society. The main themes that arose from the qualitative analysis related to Indigenous knowledges and traditions were spiritual and natural laws; ceremony and prayer; stories, histories, and original instructions; identity, gifts, and language; and ancestral knowledges, teachings, and values.

Many Elders emphasized how spiritual and natural laws are critical to guide human behaviour and align actions with the limits of the Earth. This comes from their understanding that power is not held by governments or judiciaries, but by the spirit and the land; these laws are not owned or controlled by people, but come from the spirit and the Earth itself and are non-negotiable. Several Knowledge Keepers described spiritual laws as representing the original instructions on how to live and behave properly as humans, which are expressed for example in the Anishinaabe Seven Sacred Laws and the Great Binding Law (see Oshoshko Bineshiikwe et al. 2015). Natural laws can be understood as the principles of balance that the Earth operates on, which never change and are learned through observation of the Earth, such as the law of the circle. Understandings of these spiritual and natural laws, the Knowledge Keepers explained, come from connecting to spirit and to the Earth, through ceremony, prayer, and spending time on the land. Many Elders emphasized that spiritual connection must be the first step, as it brings connection to one’s purpose, vision, and identity; each person’s spirit holds their identity, and understanding one’s own personal and collective identity is critical for living a good life. Without an understanding of identity, some participants said, people lack purpose and meaning in their lives and turn to material wealth to create meaning, thus perpetuating the environmental problems at hand. In this light, many Elders agreed that connection to identity is a critical first step, particularly for Indigenous peoples whose identities have been oppressed for so long. As Haida leader Miles Richardson explained, it is these identities that have allowed Indigenous peoples to thrive for millennia:

Our culture has guided us through living in the same place, through hundreds of generations for thousands of years. How to live the right way. How to listen so that we can understand what has been referred to as the natural law… And if we don’t comply with this law, our culture tells us, we suffer in direct proportion to our transgressions.

Indigenous identities are brought forward through stories and gifts, as well as through ancestral knowledge passed on over generations. The Elders discussed at length the rich ancestral land-based knowledges, teachings, and values developed by their Nations over thousands of years in connection with the spirit and the land. They talked about how their ancestors have lived close to and listened to the Earth, developing knowledges that are embedded within their spiritual ceremonies, languages, and their relationships to the land. Participants emphasized how it is not only the knowledge in isolation but also the ways of being and knowing in which knowledge originates and evolves, the contexts within which it is transmitted and shared, and the associated value systems, which are vitally important.

### Indigenous directions for action: connecting and collaborating

Participants emphasized that addressing climate change is urgent and that solutions must contribute to a change in values and respect Indigenous leadership in bringing forward a foundation of ancestral knowledge and traditions, which reflect a positive value system. Analysis of the discussion revealed five major themes reflecting directions for action brought forward by Summit speakers: connecting with Indigenous nationhood; connecting with spirit; connecting with tradition; connecting with the land; and connecting with each other (Table 1).

**Table 1:** Directions for action brought forward by participants of the Onjisay Aki Summit, with common themes and concepts associated with each, as identified in the content analysis

Direction for action	Themes	Main concepts
Connecting with Indigenous nationhood	Indigenous leadership Sovereignty Traditional governance Trail of the Turtle Prophecy	True leaders of their homelands; duties and responsibilities of Indigenous peoples; spiritual leadership; sharing a way of life; guidance and direction; living nationhood and teachings; strong and resilient peoples; original instructions
Connecting with spirit	Identity Purpose and meaning Healing Thunderbird spirit	Spiritual guidance; prayer and faith in health; alliance and union with thunderbird spirits; gratitude; spirituality; individual purpose; spiritual poverty; memory of identity
Connecting with traditions	Traditional ways of living Collective identity	Going back to traditional ways of life; connecting with collective identity; traditional gender roles
Connecting with the land	Relationship to the Earth Guardianship stewardship Learning from land	Reliance on Earth and natural elements; love of the land; time on the land; Earth will look after us; relation to creation; caring for and protecting the land and waters; voicing for land and animals; listening to the land; the Earth as a teacher; rites of passage
Connecting with each other	Shared responsibilities Nationhood of humanity Collaboration Knowledge sharing Youth Education Celebrating diversity People power	Connecting from the heart; shared humanity; union of North and South; responsibilities to relatives; standing together; sharing across knowledge systems; reconciliation; western science and Indigenous knowledge; ancestral schools of knowledge; role of youth in society and solutions; land-based education; reforming education systems; individual action; collective action; social movements

The Knowledge Keepers and speakers emphasized that, above all, Indigenous Nations and communities must be sovereign and free to practice their traditional governance and define their own nationhood. They discussed how Indigenous peoples’ knowledge of, and roles in, maintaining the balance of life on Earth can only be fully expressed within traditional governance systems. As sovereign Nations, communities would operate alongside nation states and have control over if and how they would engage with these states. There were differences in opinion among participants in advocating which pathways to freedom and self-determination in governance should be pursued; some talked about working to reform, replace, or abolish the Canadian Indian Act and other colonial legislations governing Indigenous peoples, while others emphasized the importance of turning away from the Indian Act and ‘breathing life into sovereignty’. There was extensive discussion around nationhood, and encouragement of Nations to define and assert their traditional laws and systems in their territories. As an example, Miles Richardson talked about the process the Haida Nation has taken to define and uphold their nationhood and jurisdiction over their territory.

Through the articulation and embodiment of their nationhood, Indigenous peoples reclaim positions of leadership to help guide humanity in returning to a way of life in balance with the Earth’s limits and laws. Anishinaabe Elders at the gathering described this as the ‘trail of the turtle’, which is understood as ‘a path that will guide humanity back to a way of life based on traditional values and balance’, led by the spirit of the grandmother turtle which represents truth. The Knowledge Keepers also talked about many Indigenous prophecies that have foretold of Indigenous leadership in this time of environmental transformation, as Elder Dr. Dave Courchene described:

Through the prophecies of our people it was said that the day would come that we would be offering and sharing a knowledge that would help all of us to come to terms again with our behaviour. And in the prophecy, we were encouraged to take our rightful place of leadership in our homeland.

From a foundation of defining and living their nationhood, the discussion focused on actions to connect with culture and tradition, beginning with spirit. Participants discussed how connection to spirit can provide individual healing, a sense of purpose, and gratitude, which are understood as prerequisites for humanity to heal our relationship with the Earth. Dene Knowledge Keeper and former Premier of the Northwest Territories, Stephen Kakfwi, shared a lesson from his Elders on the importance of spiritual connection: ‘If you don’t have a spiritual connection, and an appreciation for how you relate to everybody, you’re empty, you’re a machine’. Elder Dr. Dave Courchene emphasized that spirit is common in all people, in all living beings, which gives the identity of that life; it is each individual’s responsibility to understand and connect with their spirit and identity to guide them.

Many participants talked about the legacies of colonialism which have instilled fear and negativity around traditional ways of life (those which communities had prior to European influences and disruption through colonization) and how this must be challenged and overcome. They said Indigenous communities, particularly youth, should be encouraged to decolonize and embrace their culture as a source of strength. Anishinaabe Grandmother Florence Paynter said, ‘I think sometimes we are faced with these challenges to reinforce our own way of being, and to reaffirm that our own way of being is going to be the way to go. Going back to our own spirituality, and going back to the things that really mattered to us as people’. Several participants also talked about actions to reconnect and revitalize traditional roles and responsibilities, including recognizing the leadership of women.

Through this cultural and spiritual connection, participants discussed the importance of Indigenous peoples fostering their ancestral knowledges and connection with the Earth. Knowledge transmission between Indigenous Elders and youth was a common theme, and the idea was brought forward to develop ‘Ancestral Centres of Knowledge’ to provide opportunities for land-based education for intergenerational knowledge exchange. It was reiterated that connection to ancestral knowledge comes through connecting to the land, and thus, spending time out on the land is a key part of the solution. Participants shared that spending time on the land can bring a deeper understanding of traditional values and teachings; teach people how to listen to the messages of the Earth; connect people to their Indigenous languages; and allow individuals to develop a deeper connection with spirit.

Guardianship and stewardship were common themes, an understanding that everything that the Earth provides comes with responsibilities to care for. One example of an action to strengthen connection to the Earth and nationhood which was discussed was the Indigenous Guardians Programs.^1^ These programs are created to teach young people how to be true stewards of the land, fostering knowledge transmission while providing sustainable employment options. Japanese Knowledge Keeper and shaman Yoshimaru Higa explained ‘we are present on this Earth as guardians’, while Elder Dr. Dave Courchene described an understanding of guardianship ‘not [as] protecting the Earth, but listening to the Earth and bringing forward her messages’. For Indigenous peoples who have been displaced from their ancestral lands, the Elders emphasized that they can make a connection to the land anywhere, and are encouraged to travel to sacred sites where possible as places of significant spiritual connection.

While many Indigenous peoples maintain strong ancestral connections to the Earth, participants emphasized that responsibilities of stewardship are shared among all people that have come to their homelands. In this light, the Elders discussed the need for knowledge sharing, relationship building, and collaboration across cultures and geographies. There was a specific focus on the need to bring together Indigenous knowledge and western science on climate change, in a way that recognizes both as equally valuable and illuminate both the commonalities and distinctions between the different knowledge systems. As Dene storyteller Lawrence Nayally shared ‘There’s still so much incredible knowledge that our people have that the world has never heard before’. Plains Cree and Anishinaabe Knowledge Keeper Alvin Manitopyes acknowledged that there is increasing recognition of Indigenous wisdom around the world: ‘We have suffered so much, but yet, we have so much to give to the world, and the world is starting to recognize the validity of our spiritual knowledge’.

Building relationships across cultures was discussed as important not only for knowledge sharing, but also as a way to combat the mindset of division and competition created by colonialism. Participants discussed the need to build a movement towards defining a ‘nationhood of humanity’—uniting the human family while also celebrating the diversity of the contributions and gifts of different peoples. Several speakers compared the diversity of the human family to the diversity found in nature, something that should be recognized as beautiful and a source of strength. Miles Richardson described how this diversity can strengthen humanity’s resolve through the challenges faced:

One of my old chiefs, Chief Skidegate Lewis Collinson said, “Our forests are made up of trees. Much like the people who populate this Earth, each person and each tree is different. Different colours, different faiths, different beliefs; they come from different places. But like the forests of our islands… when troubles come to us, they come to us all.” And if we are going to withstand the winds of those troubles, like the forests we must intertwine our roots so strongly that these winds of our troubles cannot blow us over.

The Summit speakers underscored the power that all people can have individually and collectively to bring about change, and the urgency and magnitude of the actions needed. On the final day of the Summit, participants drafted and adopted the *Onjisay Aki International Climate Calls to Action*, which outlines direction for actions to strengthen ancestral knowledge, sovereignty, relationships, and transformation.^2^ Elder Dr. Dave Courchene described those who will lead this movement of values-based social change as ‘the people of the heart’. Participants talked about the need for positive, diverse solutions, starting on a personal level, rooted in spiritual and relational responsibilities. Katherine Whitecloud emphasized this shared responsibility:

We are part of this generation, this era that has created this catastrophe. And that is what it is, it is a catastrophe. Therefore, it is our responsibility, every day, because we only have today. Only Creator knows if we are going to wake up tomorrow morning. We have today, to do something about it.

**Fig. 2:**
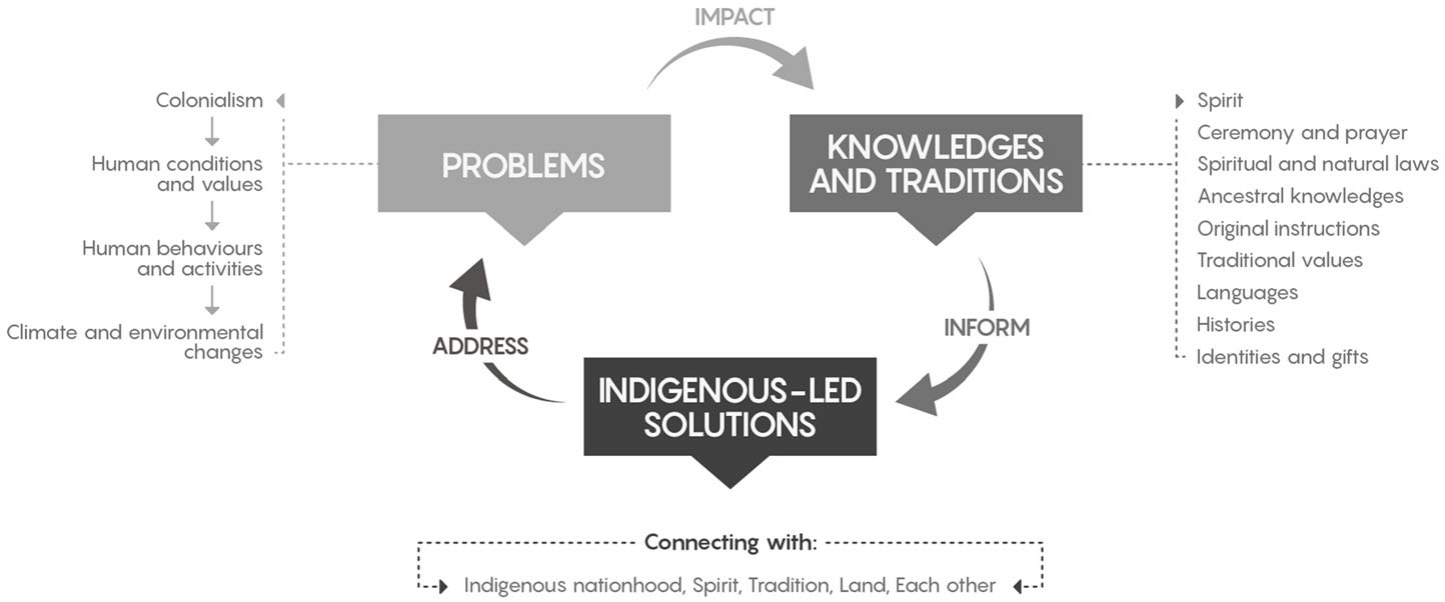
The major themes from analysis of the Onjisay Aki Summit discussions. Participants discussed the problems underlying climate change, which have impacted their knowledges and traditions. At the same time, the latter were considered sources of strength to inform solutions to climate change that address the underlying societal problems

Overall, speakers shared perspectives on the causes, impacts, and solutions of climate change, illustrating a deep and interconnected understanding of the issue and an expansive approach to solutions, rooted in ancestral knowledges, traditions, and relationship (Fig. 2).

Several short videos were made with footage from the Summit to share the spirit of the Onjisay Aki gathering and some of the highlights from the discussion with a wider audience (e.g. https://www.youtube.com/watch?v=xqPKCQCcjiU&t). A website was also created to share these videos, along with the Calls to Action, information about the speakers, and outcomes of subsequent gatherings (www.onjisay-aki.org).

## Discussion

The Onjisay Aki Summit brought forward high level yet deep understandings of the problems associated with climate change from diverse Indigenous perspectives across the Americas. The results provide insights for academic and political discourses, deepening understandings of Indigenous knowledge on climate change, as well as the ways in which this knowledge is gathered, documented, and shared through the leadership of the Knowledge Keepers. These contributions to both content and process are discussed in the following sections.

### Contributions to content: climate change and the human condition

The speakers at Onjisay Aki individually and collectively contributed understandings of the problems underlying climate change, direction for actions to address these problems, and the role of Indigenous peoples and their knowledges in these solutions. For the participants, climate change is not an external, isolated problem or natural phenomenon, but is a human, societal, and deeply personal challenge. These understanding and description of the fundamental problem underlying climate change—the human condition resulting from disconnection from land and spirit due to colonialism—contribute to the growing body of work aimed at reframing climate change from Indigenous perspectives (e.g. McGregor 2018; Reo and Parker 2013; Whyte 2017a, 2018).

Given these understandings of the problems underlying climate change, the Knowledge Keepers made clear that climate change cannot be addressed without simultaneously addressing the legacies and realities of historic and ongoing colonialism, and its paradigm of domination. In order for people to reconnect with the land, as suggested by Onjisay Aki participants, the contexts of settler colonialism and displacement of Indigenous peoples from their lands must be recognized and addressed. Efforts to shed light on historical displacement and a path forward for reinstating Indigenous jurisdiction in Canada have been gaining traction in recent years through scholarship and activism popularizing the movement for ‘land back’ (Yellowhead Institute 2019).

While calls from Indigenous scholars to recognize the colonial origins and contexts of climate change are growing (e.g. see Dhillon 2018), colonialism is often not extensively engaged in the literature on IK and climate change in Canada. Cameron (2012) reviews the literature documenting the human dimensions of climate change in the Arctic involving Inuit peoples, and finds that the majority of studies do not discuss the role and influence of colonialism on research subjects, objects, findings, and research relations, or the significance of resource exploration, extraction, and shipping as human dimensions of climate change. She argues that this exclusion of colonial and postcolonial influences in the Arctic literature risks re-inscribing colonial relations by ‘buttressing political and intellectual formations that underwrite a new round of dispossession and accumulation in the region’ (p. 104). At the international level, Ford et al. (2016) review the Fifth Assessment Report of the International Panel on Climate Change (IPCC) Working Group II and reveal that histories of colonialism, oppression, and/or racism are only found in two paragraphs out of thirty chapters. More generally, they found that content relating to Indigenous peoples ‘primarily focuses on the proximate factors affecting impacts, adaptation, and vulnerability (for example, poverty, ill health, changing livelihoods, marginalization and the erosion of TK) without posing the deeper questions around why these conditions exist, and the historic, political, social, and economic processes that have led to them’ (p. 351). This reflects a broader tendency among some governments, academics, and organizations to depoliticize the problem of climate change: to divorce the social, political, historical, and cultural contexts from the problems and impacts, framing the problem as separate from society rather than a result of societal conditions. This approach is problematic in part due to its role in limiting the ability to link action on climate change to broader goals or processes of decolonization (Ford et al. 2016). The discussion at Onjisay Aki echoes previous calls to foreground colonial contexts and dynamics in research and policy on climate change and the importance of decolonization as a multifaceted climate solution that supports healthy communities, environments, and atmospheres.

Related to the under-engagement with colonial contexts in research are considerations around the level of engagement with and representations of Indigenous knowledge systems and ways of knowing. Participants at the Onjisay Aki Summit illustrated the importance of engaging with IK beyond its local and environmental dimensions, to look at the underlying value systems and ways of knowing. While there has been significant progress made in including Indigenous peoples and their knowledges in policy and academic arenas on environmental change over the last four decades, IK is still often regarded as a source of empirical observations rather than complex, dynamic, living systems with distinct axiologies, epistemologies, and ontologies (Watson and Huntington 2014). This may be, in part, reflective of academia’s tendency to fragment knowledge into disciplines and to formulate research through the assumptions of enlightenment thought, failing to sufficiently engage non-western subjectivities (Aporta and MacDonald 2011; Watson and Huntington 2014), in this case IK of Indigenous peoples in the Americas. Increasingly, Indigenous and allied scholars and leaders are pushing back on this legacy of information extraction and erasure and charting a different course for climate research, one led by and for Indigenous communities to support adaptation capacities (Whyte 2017a). For example, the recent US National Climate Assessment has a chapter dedicated to impacts in Indigenous communities that was authored predominantly by Indigenous leaders and scholars (Jantarasami et al. 2018). From projects such as Rising Voices: Climate Resilience through Indigenous and Earth Sciences (risingvoices.ucar.edu) and Indigenous Climate Hub (indigenousclimatehub.ca), to special issues focused on Indigenous peoples’ experiences of climate change (Maldonado et al. 2016), a new standard is being set for ethical and effective approaches to research related to Indigenous perspectives on climate change. Onjisay Aki participants underlined the importance of Indigenous and non-Indigenous peoples gaining a deeper understanding of the values, philosophies, and ways of life in which IK is embedded, in order for research to accurately reflect and be shaped by Indigenous communities.

Considering that IK is embodied—that gaining knowledge is not only a matter of understanding Indigenous peoples’ relationships to the natural world and all of creation, but is the relationship itself (McGregor 2004, 2013)—it follows that Indigenous peoples must be leading research involving their knowledges to ensure all dimensions of their knowledge systems are included. The benefits of Indigenous-led research processes on climate change are twofold: they allow deeper, more multi-dimensional understandings of IK on climate change to be documented than are feasible within western-led research frameworks, while also embodying principles of self-determination and playing a role in reversing the colonial dynamics at the root of climate change. The Onjisay Aki Initiative as an example of an Indigenous-led climate research process provides important insight and lessons learned for research in this field, as discussed in the next section.

### Contributions to process: a model for Indigenous-led research

The Onjisay Aki Summit exemplified a process of Indigenous-led research on climate change, which is part of a growing movement to shift from community-based to community-led research in Indigenous contexts (Coombes et al. 2014). The approach carried out by Turtle Lodge and their collaborators reverses conventional research dynamics: every step of the initiative and process was led by the Turtle Lodge community (the Lodge Keeper, resident Knowledge Keepers, and supporters) and designed to suit their goals and needs, while their academic collaborators were invited to serve a supporting role in helping document, synthesize, and share the knowledge with a wider audience (and this paper is a reflection of that). This illustrates the broader shift in the field from how to best ‘involve’ Indigenous peoples and ‘engage’ with their knowledges in climate change research to discussing how Indigenous Knowledge Keepers and communities themselves are defining and leading collaborative research and action on climate change. While the portrayals of Indigenous peoples in the literature on climate change have started to shift in recent decades—from passive victims to active participants and contributors to solutions (Martello 2008; Raygorodetsky 2011)—the Onjisay Aki Initiative echoes calls for a further shift to recognizing Indigenous peoples as leaders and the original stewards. Central to this process is Indigenous communities’ control over every element of the research process, from setting objectives to allocating funds to shaping the narrative around results and implications. In order for this process to be truly separate from state systems and institutions, there must be a foundation of traditional governance, rooted in a ceremonial context, which guides the processes and outcomes.

#### Ceremonial context and traditional leadership

The traditional setting of the Lodge, the ceremonial context, and the leadership of the Knowledge Keepers in guiding discussion were central to the sharing process in the Summit, and reflective of ancient practices of Anishinaabe traditional governance. Ceremony invited participants to connect with the spirit and ancestors who provide knowledge, guidance, and deeper understanding of the problems and the path forward. Participants shared and learned why a spiritual connection is so important to Indigenous Knowledge Keepers and peoples, the spiritual realm being what inspires the dreams and visions that offer direction. Each day began with Pipe and Water ceremonies, including a special Pipe ceremony with a Sacred Pipe commissioned by a national group of Elders from the four directions across Canada (see Turtle Lodge 2017). Also, a Thunderbird ceremony was a central part of the Summit; the Thunderbirds are understood as one of the spiritual guardians and protectors of the Earth, and these ceremonies help people align with the Thunderbird spirits to support the natural laws of the Earth. In following their prophecies, an Eagle and Condor ceremony was also held in the evening to celebrate the union of the spirits of Indigenous peoples of the Eagle in North America and of the Condor in South America.^3^ This union was also honoured in an Inca ceremony to plant corn seeds from Peru at the Turtle Lodge, a symbol of alliance and sharing between Indigenous Nations in the Americas that modelled a way of acting in accordance with values of giving and sharing integral to human survival. On the final day of the Summit, the Onjisay Aki Calls to Action document was taken into ceremony to be spiritualized in the river and carried around the world through the waters. These ceremonies were foundational in opening and guiding the discussions and outputs of the Summit.

Considering that Indigenous spirituality has been subject to destruction and appropriation through colonization, maintaining Indigenous spiritual practices and knowledges is an act of resistance (Pettipas 1994; Smith 1999). The ceremonial process and context of knowledge sharing is in this case better suited to Indigenous knowledge systems and protocols around knowledge transmission, and may allow more and/or different information to be shared than if the gathering was held in a western context. In particular, maintaining the ceremonial context of knowledge transmission may allow the spiritual dimensions of Indigenous knowledges to be more fully included. The Elders emphasized the spiritual part of the Summit process as the first priority, understanding the ways of knowing to be just as important as the knowledge outcomes themselves.

#### Cross-cultural collaboration

Onjisay Aki exemplified the type of collaboration across cultures and geographies that participants suggest is critical in addressing environmental challenges. The Summit brought together Knowledge Keepers from Indigenous Nations across Turtle Island and the Americas—highlighting common principles, values, and observations, while providing opportunities to discuss differences and learn from one another. This initiative could be considered what scholar Salaita (2016) terms inter/nationalism—a type of decolonial thought and practice extending beyond colonial nation-state borders, thereby rejecting settler nationalism and connecting and strengthening the struggles of colonized Nations. By bringing together Knowledge Keepers from diverse Nations, Onjisay Aki also combats the exclusively ‘local’ and ‘place-based’ framing of Indigenous peoples and their knowledges in the context of climate change (Watson and Huntington 2014), particularly given the context of displacement and dispossession of land that many Indigenous peoples have faced (Gray 1995). While many research studies work with one community or region due to feasibility and/or to avoid pan-Indigenous framing, Onjisay Aki provided a unique opportunity for documenting knowledges across cultures and geographies that is reflective of the reality of communities’ collaborations across Indigenous Nations and state borders. Additionally, the participation of non-Indigenous participants and witnesses was recognized as important for cross-cultural dialogue and knowledge exchange, relationship building across diversity, and opportunities to receive guidance from the original stewards of the lands.

#### Limitations of the process

While the Summit provided a unique and valuable opportunity for knowledge sharing, there were some limitations of the process from the perspective of research, and lessons learned for future studies. Sharing knowledge in a talking circle in the Turtle Lodge reflects the oral tradition of many Indigenous Nations; however, this knowledge is distanced to a degree from the context in which it was originally shared to be documented here in writing, and is brought into conversation with academic literature. Thus, the approach is not entirely exempt of the knowledge isolation and fragmentation within academic processes that we critique herein. Participatory video methods were used to mitigate some of these challenges, since these visual approaches honour the embodied knowledge, voices, and oral histories of participants and present their knowledges in a more accessible and contextualized form. At the same time, video methods have their own considerations and limitations, as the video editor maintains a significant amount of power and control over the narrative (Bali and Kofinas 2014; Evans and Foster 2009). Another limitation arose around language; understanding of the knowledge shared in Knowledge Keepers’ native languages was limited to those at the gathering who knew the language, and was only included in the present analysis to the extent that it was translated by the Knowledge Keepers while they were speaking. Ability to translate was limited by time, resources, and practicality, due in part to the many different languages of participants. While most of the discussion took place in English, many Elders emphasized that the process of speaking in English greatly limits the knowledge that can be shared. With these considerations, the results shared here reflect only part of the discussion, and not the full depth and dimensions of the stories and knowledges shared across Nations. The challenges associated with respectfully and collaboratively documenting and communicating IK in the context of climate and environmental research have been well-documented (e.g. Louis 2007; Mistry and Berardi 2012, 2016; Williams and Hardison 2013). The present study is not immune to these challenges, and recognizes that, while this Indigenous-led and community-based research strived to meaningfully engage with Indigenous epistemologies, it is still informed and perhaps limited by the western and academic approach to ‘writing up’ research results for a mostly scholarly audience. However, given our diverse approach to sharing the outcomes of Onjisay Aki—from in-person gatherings and calls to action to video and peer-reviewed journal articles—we believe we have found a multifaceted process to maximize the impact of the project that honours different ways of knowing.

## Conclusion

Literature on Indigenous knowledges and perspectives on climate change has grown within the human dimensions of climate change field in Canada, and along with it a critical discourse on the framing of Indigenous peoples and their knowledges, the colonial contexts and dynamics of research, and the epistemological differences in knowledge systems. New insights into perspectives and process in this field have been brought forward by Turtle Lodge’s Onjisay Aki Initiative, addressing many of these critiques by creating a unique opportunity for knowledge sharing and research on climate change with Knowledge Keepers from Nations across North and South America. The discussion at the Onjisay Aki Summit emphasized an understanding that climate change is a symptom of a problem with the human condition and values, and thus, we must engage Indigenous knowledge systems in their entirety—particularly the underpinning values, spiritual dimensions, and philosophical bases—to bring about a societal shift and better understand directions for action. The solutions brought forward were rooted in Indigenous nationhood and self-determination, supporting connection with their traditions, cultures, knowledges, and lands that western activities and ideologies have physically, mentally, and emotionally aimed to displace and/or destroy. From this foundation, participants emphasized the importance of collaborative actions among people from all backgrounds to connect to the land and spirit, and share knowledges to address the larger injustices underlying climate change. However, given that many Indigenous peoples have been forcefully displaced from their ancestral homelands through colonization, with those lands now occupied by settlers, the project of reinstating those connections to land in settler colonial contexts is not straight forward.

The holistic, interconnected perspectives of the social and economic systems driving climate change that were brought forward at the Summit offer insights for action and research. In stark contrast to the framing of climate change as an issue of the environment, or one that can be addressed through technical and technological intervention, the Elders contend that it is principally social change that is needed—‘a change of the heart’, rooted in the spirit. This aligns with previous work from Indigenous and allied scholars, and suggests that actions should be taken not only to address the problem at its surface (for example by replacing conventional with alternative energy sources to reduce greenhouse gas emissions) but also to shift societal values and behaviours underlying the problems (for example by supporting land-based training and education to decrease use of and reliance on external energy sources and materials).

Furthermore, emphasizing the inherent and causal link between colonialism and climate change reiterates the need to find ways of doing research and documenting IK on climate change that reject colonial dynamics and characterizations of Indigenous peoples. By taking this approach, the Elders and Knowledge Keepers help us to see climate change not as a technical and scientific issue—focused on greenhouse gas emissions exclusively—but rather a deeply human story about the relationality between humans and the planet, and the consequences associated with colonial ideologies and actions that subjugate humans, ecosystems, and ultimately planetary balance. With increasing interest in—and funding support for—research in this field, it is a vital time to think critically about research methods and approaches. As is argued herein, the Onjisay Aki Initiative provides new insights for research processes led by and for Indigenous peoples, and helps build on this evolving movement and associated literature. A foundational element of this process is traditional governance, rooted in ceremonial context and led by Knowledge Keepers. In this respect, the project raises important questions for future research, such as the following: What is and is not considered research on climate change? What language is being used to discuss climatic and environmental changes, and how does this interface with different knowledge systems? What venues for knowledge sharing are most effective in receiving, learning, and understanding Indigenous knowledge? How can ceremonial contexts be honoured and engaged as knowledge sources in research processes? Based on the results shared here as well as those from previous work in the field, we suggest several principles to be particularly mindful of in research and knowledge sharing processes in this field: (1) to respect, follow, and engage with ceremonial protocols and contexts associated with knowledge sharing in Indigenous communities; (2) to acknowledge IK as complex and dynamic systems and processes, and to strive to engage the epistemological and ontological dimensions; (3) to recognize IK as equally valid and valuable alongside western scientific knowledge; (4) to be and remain flexible as researchers and institutions in research approaches, striving for inter-epistemological research that allows the process to be influenced and guided by non-western ways of knowing where possible.

### Supplementary information

ESM 1 (MP4 398,801 kb)

## Appendix. Participants of the Onjisay Aki International Climate Summit

- Dr. Dave Courchene (Nii Gaani Aki Inini – Leading Earth Man)*, Knowledge Keeper of the Anishinaabe Nation, Founder of Turtle Lodge, Convener of the Onjisay Aki International Climate Summit
- Katherine Whitecloud*, Knowledge Keeper and Dakota Nation Spokesperson
- Paul K. Chappell, Peace Leadership Director, Nuclear Age Peace Foundation
- Deanna Pashe (Taksha’ Pu’juta Win)*, Dakota and Anishinaabe Nations, Assembly of First Nations Youth Council Senior Executive
- Bill McKibben, Founder of 350.org
- Chief Jack Caesar (Nei Kau Da Zo)*, Knowledge Keeper and Chief of Ross River Dene Nation
- Alvin Manitopyes*, Knowledge Keeper of the Plains Cree and Anishinaabe Nations
- Florence Paynter*, Knowledge Keeper of the Anishinaabe Nation
- Stephen Kakfwi*, Dene Nation, Former President of the Dene Nation and Former Premier of the Northwest Territories
- Chief Arvol Looking Horse*, Knowledge Keeper and Spiritual Leader of the Dakota, Lakota and Nakota Nation
- Inka Antaurko*, Knowledge Keeper and Spiritual Leader of the Inka Amauta, Peru
- Alexander Kofi*, West African, Blackfoot Lakota and Cherokee/Choctaw Nations, Athlete, Reggae Artist, Activist, USA
- Scott Vaughan, President and CEO, International Institute for Sustainable Development
- Miles Richardson*, Haida Nation, Former President of the Council of the Haida Nation
- Jeewan Chanicka, Superintendent of Equity, Anti-Racism and Anti-Oppression, Toronto District School Board
- Everton Gordon, Interim CEO of the Jamaican Canadian Association (JCA); Executive Director of Caribbean African Canadian (CAFCAN) Social Services, Canada
- Elmer Courchene*, Knowledge Keeper of the Anishinaabe Nation, Lead Elder of the Assembly of First Nations Elders’ Council
- Yoshimaru Higa (Kumiko Ahara)*, Knowledge Keeper, Shaman and Spiritual Leader, Japan
- Lawrence Nayally*, Dene Nation, Host of Trails End, CBC News
- Vivian Delgado*, Knowledge Keeper of the Tewa Pueblo and Yaqui Pueblo Nation from Taos Territory
- Paqarina Wanka (Sonia Astuhuaman)*, Knowledge Keeper of the Inka Nation, Andean Medicine Woman
- T’ito Kuntur-Kanki (Rodolfo Ttito Condori)*, Knowledge Keeper of the Inka Nation, Member of the Andean Masters Community

*Indigenous participants

## Footnotes

1 https://www.ilinationhood.ca/our-work/guardians/

2 Available at http://onjisay-aki.org/onjisay-aki-international-climate-calls-action

3 To read more and watch a video about the prophecy, see http://onjisay-aki.org/prophecy-eagle-and-condor
